# Nomogram for predicting coronary artery lesions in patients with Kawasaki disease

**DOI:** 10.1002/clc.24113

**Published:** 2023-08-04

**Authors:** Jie Chen, Jing Li, Yang‐hua Yue, Yu Liu, Tian Xie, Jian‐qiao Peng, Zhong‐hua Deng, You‐de Cao

**Affiliations:** ^1^ Department of Clinical Laboratory, Hunan Provincial People's Hospital The First Affiliated Hospital of Hunan Normal University Changsha China; ^2^ Department of Infectious Diseases Youxian People's Hospital Zhuzhou China; ^3^ Department of Pediatrics, Hunan Provincial People's Hospital The First Affiliated Hospital of Hunan Normal University Changsha China

**Keywords:** coronary artery lesions, Kawasaki disease, nomogram, risk factor

## Abstract

**Background:**

Coronary artery lesions are the most important complications of Kawasaki disease. Approximately 25–30% of untreated patients develop coronary artery disease, which can lead to long‐term cardiovascular sequelae.

**Aim:**

The aim of this study is to evaluate the risk factors for coronary artery lesions in Kawasaki disease and to construct a nomogram for predicting the likelihood of developing such lesions.

**Methods:**

Data from 599 patients between January 2012 and June 2020 were reviewed retrospectively. Patients were randomly assigned to the training set (*n* = 450) and the validation set (*n* = 149). A comparison of clinical features and laboratory data was performed, followed by multivariate logistic regression analysis to identify independent risk factors and develop the nomogram. The predictive efficiency of the nomogram was evaluated using the calibration curve, area under the receiver operating characteristic curve (AUC), C‐index, and decision curve analysis (DCA).

**Results:**

Intravenous immunoglobulin (IVIG) resistance, delayed IVIG treatment, C‐reactive protein, and neutrophil/lymphocyte ratio were identified as independent risk factors for the development of coronary artery lesions. The nomogram was constructed based on these four variables. The calibration curve of the nomogram showed a high degree of agreement between the predicted probability and the actual probability. The AUC of the nomogram in the training and validation set was 0.790 and 0.711, respectively. In addition, DCA revealed that the nomogram provided a significant net benefit, further supporting its clinical utility.

**Conclusions:**

The constructed nomogram demonstrates a strong and reliable performance in predicting coronary artery lesions, which enables clinicians to make timely and tailored clinical decisions.

## Graphical abstract

The criteria of current scoring systems for predicting coronary artery lesions are not fully consistent and the prediction efficiency varies among different populations. Tools to predict coronary artery lesions are still essential. In our study, intravenous immunoglobulin (IVIG) resistance, delayed IVIG treatment, C‐reactive protein and neutrophil/lymphocyte ratio were identified as independent risk factors for coronary artery lesions. The constructed nomogram based on these four variables demonstrates high sensitivity, high specificity, and high efficacy in predicting coronary artery lesions in patients with Kawasaki disease.

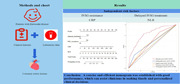

## 1 INTRODUCTION

Kawasaki disease (KD) is a systemic vasculitis of unknown etiology that predominantly affects in children under 5 years of age. The primary pathological feature involves extensive inflammation of small to medium‐sized blood vessels, as well as various tissues and organs.^1^, ^2^ Coronary artery lesions (CAL) are the most important complications of KD, including coronary dilatation and aneurysms, which are primarily associated with coronary artery stenosis, ischemic heart disease, and even cardiac death.^3^, ^4^ Consequently, the quality of life for affected children is profoundly compromised and their lives are even endangered. During the acute stage, 5% ~ 20% of patients with KD developed CAL despite receiving high‐dose intravenous immunoglobulin (IVIG) treatment.^5^, ^6^, ^7^ As a result, KD has become the most frequent acquired heart disease in pediatrics.

The incidence rate of KD has been increasing in recent years.^8^ Moreover, coronary artery aneurysm (CAA) in KD has received increasing attention.^9^, ^10^ However, the majority of CAL in KD are coronary artery dilatation, not CAA.^11^, ^12^ Early identification and timely intervention of KD patients at high risk of CAL are key points for improving the prognosis. To date, approximately 10 scoring systems have been developed for predicting the risk of CAL. Nevertheless, the criteria used in these scoring systems are not fully consistent with previous findings, and the predictive efficiency varies across different populations. For instance, Kobayashi scoring system was recently used to evaluate CAA risks in German populations,^13^ resulting in a sensitivity was 53% at a cut‐off of three, which is considerably lower than that in Japanese populations (90.6%). Kobayashi, Egami, and Formosa scoring systems were applied to predict CAL in Italian cohort and the predictive efficacy of the three scoring systems was poor.^14^ Furthermore, the evaluation of eight scoring systems for predicting CAL in Chinese populations revealed a range of sensitivity from 5.88% to 52.94% and the specificity from 41.94% to 94.62%.^15^ Hence, a reliable and user‐friendly scoring system for accurately predicting the risk of CAL remains imperative. In contemporary medical practice, nomograms are widely applied for diagnosing and predicting disease onset or progression, wherein each parameter is assigned a corresponding risk score. The present study is designed to evaluate the risk factors associated with CAL in patients with KD and establish a nomogram to facilitate its prediction.

## 2 MATERIAL AND METHODS

### 2.1 Inclusion and exclusion criteria

All KD patients admitted to Hunan Provincial People's Hospital between January 2012 and June 2020 were eligible for inclusion. The diagnostic criteria for KD were based on the American Heart Association (AHA) guidelines.^1^ Patients were diagnosed with complete KD when they had a high fever (≥39°C) lasting for at least 5 days and presented with ≥4 of the following five major clinical manifestations: (1) bilateral conjunctive injection; (2) polymorphous rash; (3) oral mucosal changes; (4) changes in the peripheral extremities; (5) cervical nonsuppurative lymphadenopathy. Patients with a high fever for ≥5 days and with only two or three major clinical symptoms were diagnosed as incomplete KD, while no other possible causes of fever could explain the illness. Exclusion criteria included the following: (1) suspected KD; (2) not in the acute stage; (3) received IVIG at another hospital; (4) CAL were diagnosed before IVIG treatment; (5) with a history of KD and cardiovascular system diseases; (6) concomitant with metabolic diseases, tumors and/or blood system diseases; (7) incomplete medical records.

Transthoracic 2‐dimensional echocardiography was performed by experienced pediatric echocardiographers during the acute stage of KD. The diagnosis of CAL was in accordance with the Japanese Ministry of Health (JMH) criteria: the coronary artery internal lumen diameter ≥3 mm in children <5 years of age or ≥4 mm in children ≥5 years of age, the coronary artery diameter exceeds 1.5 times the diameter of the adjacent segment, or the coronary artery lumen is clearly irregular.

All patients received IVIG (2 g/kg given as a single intravenous infusion or 1 g/kg per day for 2 consecutive days) and oral aspirin (30–50 mg/kg/day), and aspirin was gradually reduced to maintenance treatment dose (3 ~ 5 mg/kg) after resolution of fever and continued for 6 ~ 8 weeks. IVIG resistance was defined as persistent or recrudescent fever lasting more than 36 h after the end of the initial IVIG infusion. A second dose of IVIG was given alone or combined with corticosteroids simultaneously as additional rescue treatment. Delayed IVIG treatment was defined as the administration of IVIG after the tenth day of illness.

### 2.2 Data collection and study design

Demographic characteristics, clinical data, and the day of illness at initial IVIG administration were recorded. Laboratory data were also collected before IVIG treatment, including white blood cell count (WBC), absolute neutrophil count (N), absolute lymphocyte count (L), absolute monocyte count, platelet count, hemoglobin, hematocrit, mean red blood cell volume, mean platelet volume, total bilirubin, alanine aminotransferase, aspartate aminotransferase, γ‐glutamyl transferase (GGT), albumin (ALB), C‐reactive protein (CRP), serum sodium, serum potassium, serum chloride (Cl). Neutrophil/lymphocyte ratio (NLR), platelet/lymphocyte ratio, and CRP/ALB ratio (CAR) were calculated.

All patients in this study were randomly allocated at a 3:1 ratio to the training set and validation set. Patients were classified into the CAL and non‐CAL groups according to echocardiographic results. To establish a nomogram for predicting CAL in patients with KD, the demographic characteristics, clinical data, and laboratory data were compared between two groups in the training set. The predictive power of the nomogram was verified in the validation set.

### 2.3 Statistical analysis

Qualitative data were summarized as numbers (percentages), and the comparison between the two groups was conducted by the *χ* ^2^ test. Continuous data were described as median (interquartile ranges) and analyzed using the Mann–Whitney U test. Variables with *p* < .05 were further brought into multivariate logistic regression analysis to identify the independent risk factors for CAL. Also, the independent risk factors were combined to develop a predictive nomogram for CAL in KD. To evaluate the performance of the nomogram, internal validation was performed using the calibration curve in the training set. The discriminative ability of the nomogram was assessed using an area under the receiver operating characteristic curve (AUC) in the training and validation set. In addition, the clinical applicability and benefits of the nomogram were evaluated by the decision curve analysis (DCA). Statistical analyses were conducted using SPSS 23.0 software (SPSS, Inc.) and R software (version 4.2.1; https://www.R-project.org).

## 3 RESULTS

### 3.1 Patient characteristics

**Figure 1:**
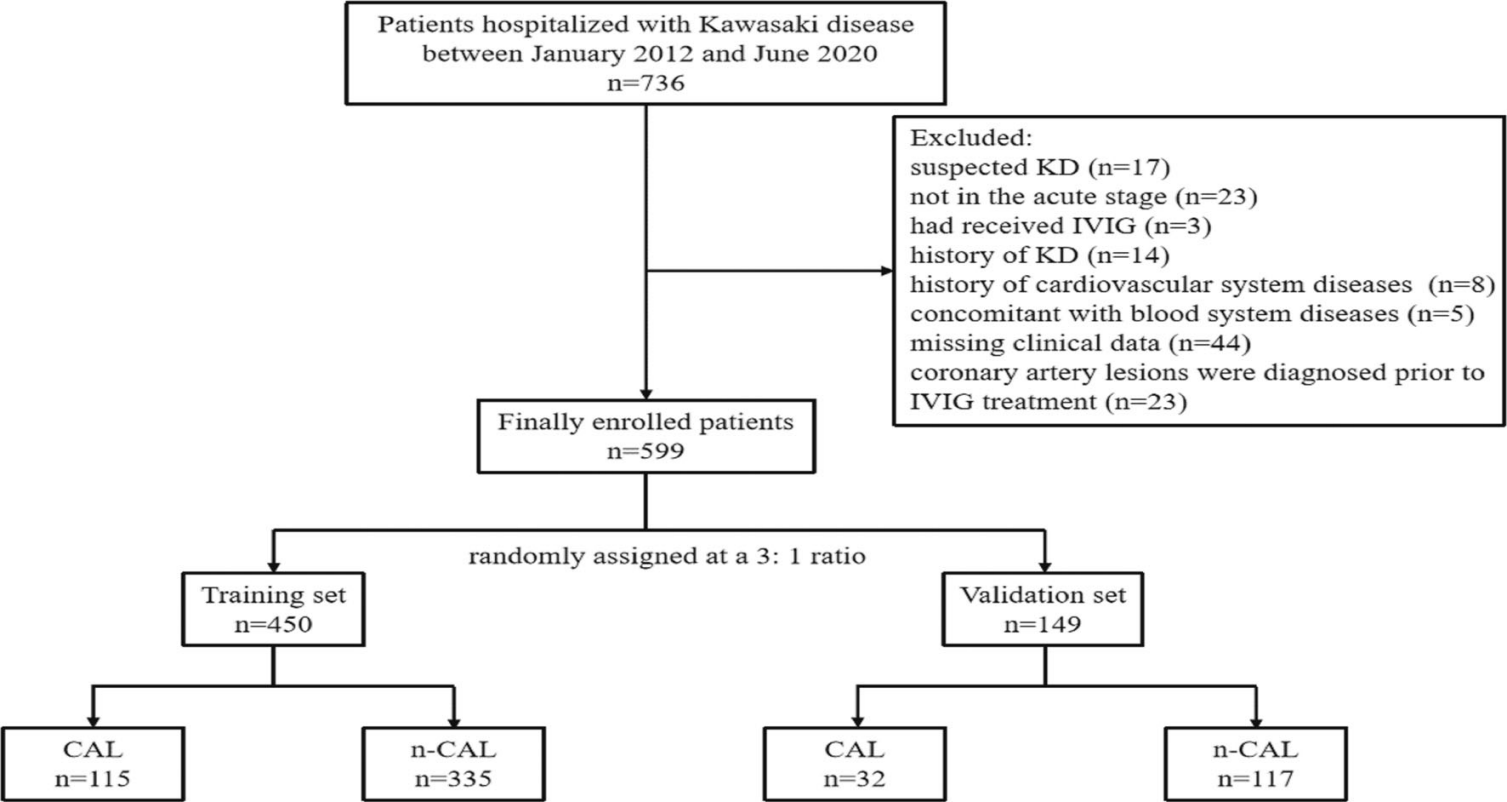
Flowchart of this study.

A total of 736 patients with KD were admitted to Hunan Provincial People's Hospital between January 2012 and June 2020, 599 patients were finally included in this study after exclusion and were randomly allocated to the training set (*n* = 450) and validation set (*n* = 149) at a 3:1 ratio (Figure 1). Supporting Information: Table S1 shows the demographic characteristics, clinical data, and laboratory data of the patients. In the primary cohort, 147 of 599 (24.5%) patients were diagnosed with CAL, while 54 (9.0%) patients were identified with IVIG resistance.

### 3.2 Screening candidate factors for predicting CAL in KD

For the training set, 115 and 335 patients were classified into the CAL group and non‐CAL group, respectively. The incidence of IVIG resistance was 21.7% (25/115) patients in the CAL group, which was significantly higher than that in the non‐CAL group (5.4%) (*p* < .05). Additionally, the proportion of patients with delayed IVIG treatment was significantly greater in CAL group than in the non‐CAL group (21.7% vs. 10.4%, *p* = .002). Univariate analysis revealed that the WBC, N, CRP, GGT, NLR, and CAR were significantly higher in the CAL group, while the L was significantly lower in the CAL group (*p* < .05). The results of the correlational analysis are summarized in Table 1. In multivariate regression analysis, IVIG resistance (odds ratio [OR] 2.575; 95% confidence interval [CI] 1.183–5.607; *p* = .017), delayed IVIG treatment (OR 2.552; 95% CI 1.312–4.965; *p* = .006), CRP (OR 1.021; 95% CI 1.006–1.036; *p* = .005), NLR (OR 1.252; 95% CI 1.082–1.449; *p* = .003) were identified as independent risk factors for CAL, as shown in Table 2.

**Table 1:** Comparison of clinical features and laboratory data between the non‐CAL and CAL group in the training set.

Characteristic	non‐CAL	CAL	*p* Value
	*n* = 335	*n* = 115	
Age (month)	22.0 (12.0–36.0)	24.0 (12.0–48.0)	.064
Sex (male/female)	222/113	76/39	.972
Clinical features			
Oral changes	308 (91.9%)	100 (87.0%)	.113
Conjunctivitis	318 (94.9%)	111 (96.5%)	.484
Extremity changes	293 (87.5%)	104 (90.4%)	.394
Lymphadenopathy	111 (33.1%)	36 (31.3%)	.718
Rash	320 (95.5%)	112 (97.4%)	.378
Incomplete KD	43 (12.8%)	20 (17.4%)	.224
IVIG resistance	18 (5.4%)	25 (21.7%)	<.001
Delayed IVIG treatment	35 (10.4%)	25 (21.7%)	.002
WBC (×10^9^/L)	12.71 (9.17–16.61)	16.11 (12.05–21.30)	<.001
N (×10^9^/L)	7.93 (5.32–11.55)	11.68 (8.13–16.75)	<.001
L (×10^9^/L)	3.08 (2.16–4.20)	2.54 (1.72–4.25)	.017
M (×10^9^/L)	0.62 (0.44–1.01)	0.65 (0.48–0.95)	.314
PLT (×10^9^/L)	374 (291–492)	369 (252–475)	.164
HGB (g/L)	105 (98–113)	106 (97–111)	.290
HCT (%)	31.7 (29.6–33.9)	32.0 (29.8–33.7)	.607
MCV (fL)	80.5 (77.3–83.4)	80.6 (77.2–83.1)	.984
MPV (fL)	9.1 (8.0–10.1)	9.2 (8.3–10.1)	.459
TB (μmol/L)	7.16 (5.20–9.80)	7.29 (5.70–11.50)	.114
ALT (U/L)	26.9 (15.5–58.7)	28.6 (15.8–70.0)	.427
AST (U/L)	34.30 (25.38–50.10)	33.53 (25.50–60.90)	.622
GGT (U/L)	19.9 (12.4–76.5)	36.7 (14.9–133.9)	.002
ALB (g/L)	37.79 (34.80–40.70)	37.70 (34.63–41.50)	.716
CRP (mg/L)	71.70 (44.00–114.00)	128.40 (75.40–160.00)	<.001
Na (mmol/L)	136.0 (134.0–137.4)	135.3 (133.9–137.2)	.199
K (mmol/L)	4.23 (3.89–4.65)	4.27 (3.91–4.60)	.881
Cl (mmol/L)	101.0 (99.0–103.0)	100.0 (98.0–103.0)	.154
NLR	2.59 (1.76–3.99)	4.50 (2.88–6.90)	<.001
PLR	124.19 (86.75–177.82)	136.20 (97.63–190.43)	.161
CAR	1.85 (1.09–3.03)	3.55 (2.04–4.46)	<.001

Abbreviations: ALB, albumin; ALT, alanine aminotransferase; AST, aspartate aminotransferase; CAL, coronary artery lesions; CAR, C‐reactive protein/albumin ratio; Cl, serum chloride; CRP, C‐reactive protein; GGT, γ‐glutamyl transferase; HCT, hematocrit; HGB, hemoglobin; IVIG, intravenous immunoglobulin; K, serum potassium; KD, Kawasaki disease; L, absolute lymphocyte count; M, absolute monocyte count; MCV, mean red blood cell volume; MPV, mean platelet volume; N, absolute neutrophil count; Na, serum sodium; NLR, neutrophil/lymphocyte ratio; PLR, platelet/lymphocyte ratio; PLT, platelet count; TB, total bilirubin; WBC, white blood cell count.

**Table 2:** Independent predictors determined by multivariate analysis for the development of the nomogram in the training set.

Variable	OR	95% CI	*p* Value
IVIG resistance	2.575	1.183–5.607	.017
Delayed IVIG treatment	2.552	1.312–4.965	.006
CRP	1.021	1.006–1.036	.005
NLR	1.252	1.082–1.449	.003

Abbreviations: CI, confidence interval; CRP, C‐reactive protein; IVIG, intravenous immunoglobulin; NLR, neutrophil/lymphocyte ratio; OR, odds ratio.

### 3.3 Nomogram development and validation

**Figure 2:**
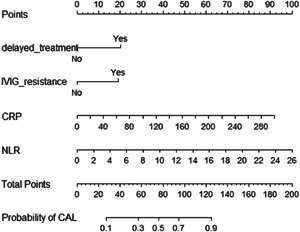
Nomogram for predicting the probability of coronary artery lesions.

**Figure 3:**
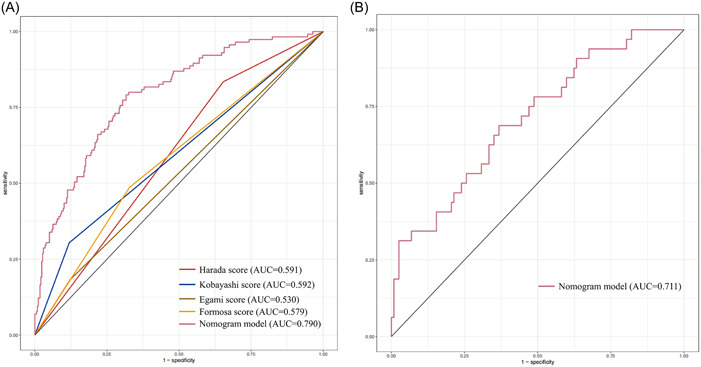
(A) ROC curves of multiple scoring systems for predicting coronary artery lesions in the training set. (B) ROC curves of the nomogram model in the validation set.

The four significant independent risk factors (IVIG resistance, delayed IVIG treatment, CRP, and NLR) identified by multivariate regression analysis were applied to develop a nomogram. The scores corresponding to these four risk factors were summed to draw a total point line for predicting the probability of CAL in KD (Figure 2). The calibration curve of the nomogram showed good agreement between the predicted probability and the actual probability (Supporting Information: Figure S1), which indicated a high level of accuracy. The AUC of the nomogram was 0.790 (95% CI 0.742–0.837), suggesting a good discrimination capacity of the nomogram (Figure 3A). For the internal validation, patients in the validation set were rated with the nomogram and calculated the AUC. The AUC of the nomogram was 0.711 (95% CI 0.610–0.811) in the validation set (Figure 3B). The C‐indices of the nomogram were 0.790 (95% CI 0.743–0.837) and 0.749 (95% CI 0.654–0.843) in the training set and validation set, respectively. Moreover, the DCA of the nomogram revealed a significant net benefit for clinical applicability (Supporting Information: Figure S2).

### 3.4 Comparisons of the nomogram model with the other scoring systems

The nomogram model contained four independent predictors, including IVIG resistance, delayed IVIG treatment, CRP, and NLR. Among them, IVIG resistance, delayed IVIG treatment, and NLR were unique to the nomogram model. The nomogram model and four other scoring systems were used to assess the CAL risk in our patients. The comparisons of the nomogram model with the other scoring systems are presented in Figure 3A and Supporting Information: Table S2. The nomogram model had a sensitivity of 79.1% and a specificity of 68.4%, while the sensitivity of four other scoring systems ranged from 18.3% to 83.5% and the specificity ranged from 34.6% to 88.1% for predicting CAL. Moreover, the positive predictive value of the nomogram model was 46.2% and the negative predictive value was 90.5%, which were higher than four other scoring systems (positive predictive values: 30.5%–46.7%, negative predictive values: 75.6%–85.9%). The AUC value of the nomogram model was 0.790, higher than Harada (0.591), Kobayashi (0.592), Egami (0.530), and Formosa scoring system (0.579).

## 4 DISCUSSIONS

KD is an acute systemic vasculitis which can lead to severe CAL, exerting a significant effect on children's health. The incidences of CAL in Shanghai,^16^ Beijing,^17^ and Hangzhou^18^ were 19.8%, 20.6%, and 24.1%, respectively. In our study, the incidence of CAL was 24.5%, which is comparable to that reported in previous studies. With the increasing prevalence of KD, it poses a considerable challenge for clinicians to treat and manage KD with CAL. Thus, early risk assessment and early diagnosis of CAL become particularly crucial. In the current study, IVIG resistance, delayed IVIG treatment, CRP, and NLR were described as significant independent risk factors for CAL in patients with KD. The predictive nomogram, based on these four factors, showed a good performance with high discrimination capacity (AUC 0.790, 95% CI 0.742–0.837). Furthermore, it was effectively validated in the validation set (AUC 0.711, 95% CI 0.610–0.811).

Prevention of CAL is the foremost aspect in KD management. High‐dose IVIG combined with acetylsalicylic acid is the first‐line treatment strategy for acute KD, effectively controlling inflammation and reducing the incidence of CAL. However, approximately 10% ~ 20% of patients fail to respond to the initial IVIG treatment and this subgroup of patients is at a higher risk of developing CAL, which may be attributed to a lack of timely intervention in the pathological process of vasculitis, resulting in a sustained severe inflammatory response.^19^, ^20^ In addition, due to the lack of specific laboratory diagnostic indicators and typical symptoms, the clinical diagnosis of KD primarily relies on a comprehensive understanding of disease manifestations, and KD is prone to misdiagnosis or missed diagnosis, which can lead to missing the optimal time for IVIG treatment. According to AHA guidelines, patients with KD should be treated with a single infusion of IVIG (2 g/kg) as early as possible within the first 10 days of illness onset. However, 10% ~ 25% of patients with KD were administered IVIG more than 10 days after illness onset.^21^, ^22^, ^23^, ^24^ In this study, IVIG resistance was observed in 21.7% of patients in the CAL group, which is similar to the findings reported by Chang et al. (22.8%),^25^ Yu et al. (16%),^26^ Hua et al. (24.1%).^18^ Muta et al.^27^ showed that patients with delayed IVIG treatment (>10 days) had an increased risk of developing CAL during the convalescent phase. Similarly, Qiu et al.^28^ explored risk factors for CAL at different time points and found that delayed IVIG treatment was correlated with CAL at 1 and 6 months after illness onset. Consistent with previous studies, our results suggested that IVIG resistance and delayed IVIG treatment were risk factors for CAL. These findings indicated that early diagnosis and treatment were crucial for the management of KD. Adjunctive therapeutic approaches, such as steroids and infliximab, may be essential for patients who exhibit resistance to IVIG.^29^, ^30^

Elevated serum CRP levels, which reflect systemic inflammation in KD, are commonly used to predict the risk of cardiovascular events.^31^ In a prospective observational study, Son et al.^10^ analyzed the risk factors for predicting CAA in a North American population, and found that Z score ≥ 2.0, age <6 months, Asian race and CRP ≥ 13 mg/dL were independent risk factors for CAA. Qiu et al.^28^ reported that delayed IVIG treatment was an independent risk factor for the development of CAL, and patients with higher CRP and ESR levels (CRP > 79 mg/L, ESR > 34 mm/h) were more likely to develop CAL. Likewise, Xie et al.^32^ reported that patients with CAL or IVIG resistance had significantly higher serum CRP levels compared to those without CAL in KD. In the initial pathological process of KD, neutrophils play an extremely important role in the initial immune response. Additionally, the release of various anti‐inflammatory cytokines triggers immunosuppression, resulting in the apoptosis of lymphocytes.^33^ Thus, the NLR may indicate the balance between inflammatory reactions and immune regulation in KD. Several studies have suggested NLR in the pathogenesis of inflammation in KD and recognized it as an independent risk factor for CAL and/or IVIG resistance.^34^, ^35^, ^36^ In accordance with these previous studies, our results demonstrated that NLR with an OR of 1.252 (95% CI 1.082–1.449) was independently associated with CAL in KD.

To date, various scoring systems have been established to predict CAL in Chinese and other populations. In our study, we evaluated the risks of CAL using a nomogram model and four other scoring systems, including Harada,^37^ Kobayashi,^38^ Egami,^39^ and Formosa^40^ scoring system. Our nomogram model exhibited a superior AUC of 0.790 compared to the four other scoring systems. In addition, our nomogram model had better sensitivity than the Kobayashi, Egami, and Formosa scoring systems but lower than the Harada scoring system. The specificity of our nomogram model was higher than the Harada and Formosa scoring system, but lower than the Kobayashi and Egami scoring system. Thus, in comparison with previous studies, our nomogram model demonstrated superior predictive capability for CAL.

There are several limitations in the present study. First, this is a single‐center retrospective study; therefore, a multicenter prospective study with a larger sample is needed to validate the model externally. Second, this study was limited by the absence of certain nonroutine testing indicators that have been reported to be associated with CAL, such as interleukin‐6 and brain natriuretic peptide. Third, it should be mentioned that CAL was diagnosed according to the JMH criteria in our study due to a large number of missing Z values.

## 5 CONCLUSIONS

In the present study, we constructed a nomogram model utilizing four clinical and laboratory parameters: IVIG resistance, delayed IVIG treatment, CRP, and NLR. Furthermore, this nomogram model demonstrates high sensitivity, high specificity, and high efficacy in predicting CAL in patients with KD. Therefore, clinicians can employ this model to quantitatively evaluate the risk of CAL, implement timely intervention management, and improve patient outcomes.

## AUTHOR CONTRIBUTIONS

Jie Chen and Jing Li were responsible for data analysis and drafting the manuscript. You‐de Cao designed the study and reviewed the manuscript. Yu Liu reviewed and revised the manuscript. Tian Xie carried out the conception and design, reviewed the manuscript, and revised it for intellectual content. Yang‐hua Yue, Jian‐qiao Peng, and Zhong‐hua Deng participated in data collection. All authors approved the final version to be published.

## Supporting information

Supporting information.

## Data Availability

The data sets generated for the current study are not publicly available due to data protection but are available from the corresponding author on reasonable request.
