# Supplementary material for: Nomogram for predicting coronary artery lesions in patients with Kawasaki disease
Source: Clin Cardiol. 2023 Aug 4;46(11):1434–41. doi: 10.1002/clc.24113 (PMC10642325; doi:10.1002/clc.24113)
Supplement: Supplementary file 1 — Supporting information. [file CLC-46-1434-s001.docx]

Table S1. Demographics, clinical characteristics and laboratory data of the KD patients.

| Characteristic | All patients | Training set | Validation set |  | |
| --- | --- | --- | --- | --- | --- |
|  | n=599 | n=450 | n=149 | P |  |
| age (month) | 23.0 (12.0-36.0) | 23.5 (12.0-36.0) | 23.0 (12.0-36.0) | 0.806 |  |
| Sex (male/female) | 396/203 | 298/152 | 98/51 | 0.920 |  |
| Clinical features |  |  |  |  |  |
| Oral changes | 546 (91.2%) | 408 (90.7%) | 138 (92.6%) | 0.467 |  |
| Conjunctivitis | 573 (95.7%) | 429 (95.3%) | 144 (96.6%) | 0.496 |  |
| Extremity changes | 523 (87.3%) | 397 (88.2%) | 126 (84.6%) | 0.245 |  |
| Lymphadenopathy | 196 (32.7%) | 147 (32.7%) | 49 (32.9%) | 0.961 |  |
| Rash | 577 (96.3%) | 432 (96.0%) | 145 (97.3%) | 0.459 |  |
| Incomplete KD | 83 (13.9%) | 63 (14.0%) | 20 (13.4%) | 0.860 |  |
| IVIG resistance | 54 (9.0%) | 43 (9.6%) | 11 (7.4%) | 0.422 |  |
| Delayed IVIG treatment | 89 (14.9%) | 60 (13.3%) | 29 (19.5%) | 0.068 |  |
| WBC (×10^9^/L) | 13.47 (9.74-17.56) | 13.66 (9.80-17.72) | 12.81 (9.57-17.33) | 0.501 |  |
| N (×10^9^/L) | 9.05 (5.69-12.55) | 9.15 (5.74-12.62) | 8.96 (5.44-12.33) | 0.547 |  |
| L (×10^9^/L) | 2.97 (1.99-4.23) | 2.98 (2.04-4.21) | 2.99 (1.95-4.23) | 0.796 |  |
| M (×10^9^/L) | 0.61 (0.44-0.97) | 0.62 (0.45-1.01) | 0.58 (0.41-0.86) | 0.075 |  |
| PLT (×10^9^/L) | 372 (278-492) | 372 (286-492) | 372 (243-508) | 0.409 |  |
| HGB (g/L) | 105 (98-112) | 105 (98-112) | 106 (99-113) | 0.470 |  |
| HCT (%) | 31.9 (29.8-33.8) | 31.8 (29.7-33.8) | 32.1 (30.3-33.8) | 0.368 |  |
| MCV (fL) | 80.6 (77.5-83.3) | 80.5 (77.3-83.4) | 80.9 (78.2-83.2) | 0.245 |  |
| MPV (fL) | 9.0 (8.0-10.0) | 9.1 (8.1-10.1) | 8.8 (7.9-9.9) | 0.139 |  |
| TB (μmol/L) | 7.23 (5.30-10.30) | 7.20 (5.30-10.20) | 7.41 (5.45-10.40) | 0.473 |  |
| ALT (U/L) | 26.5 (15.4-60.8) | 27.2 (15.5-61.3) | 24.1 (14.6-56.5) | 0.530 |  |
| AST (U/L) | 34.10 (25.62-53.69) | 34.08 (25.47-54.18) | 34.10 (26.12-52.08) | 0.734 |  |
| GGT (U/L) | 24.0 (12.6-81.9) | 25.7 (12.9-86.6) | 24.0 (12.1-64.0) | 0.454 |  |
| ALB (g/L) | 37.79 (34.70-41.00) | 37.75 (34.72-40.90) | 38.00 (34.50-41.60) | 0.651 |  |
| CRP (mg/L) | 79.50 (48.90-125.74) | 79.95 (50.38-126.00) | 76.40 (46.40-124.85) | 0.397 |  |
| Na (mmol/L) | 135.9 (134.0-137.2) | 136.0 (134.0-137.4) | 135.0 (133.7-137.0) | 0.055 |  |
| K (mmol/L) | 4.25 (3.90-4.64) | 4.24 (3.90-4.63) | 4.28 (3.88-4.69) | 0.806 |  |
| Cl (mmol/L) | 101.0 (98.6-103.0) | 101.0 (99.0-103.0) | 101.0 (98.0-103.0) | 0.945 |  |
| NLR | 2.96 (1.80-5.02) | 3.04 (1.85-4.93) | 2.71 (1.68-5.28) | 0.583 |  |
| PLR | 126.92 (88.87-185.81) | 126.51 (89.47-182.35) | 130.00 (84.64-194.07) | 0.908 |  |
| CAR | 2.10 (1.28-3.47) | 2.09 (1.39-3.55) | 2.12 (1.18-3.29) | 0.412 |  |
| CAL | 147 (24.5%) | 115 (25.6%) | 32 (21.5%) | 0.316 |  |

WBC, white blood cell count; N, absolute neutrophil count; L, absolute lymphocyte count; M, absolute monocyte count; PLT, platelet count; HGB, hemoglobin; HCT, hematocrit; MCV, mean red blood cell volume; MPV, mean platelet volume; TB, total bilirubin; ALT, alanine aminotransferase; AST, aspartate aminotransferase; GGT, γ-glutamyltransferase; ALB, albumin; CRP, C-reactive protein; Na, serum sodium; K,serum potassium; Cl, serum chloride; NLR, neutrophil/lymphocyte ratio; PLR, platelet/lymphocyte ratio; CAR, C-reactive protein/ albumin ratio; CAL, coronary artery lesions.

Table S2. Comparisons of the nomogram model with the other scoring systems in the training set.

|  | AUC | 95% CI | Sensitivity | Specificity | PPV | NPV |
| --- | --- | --- | --- | --- | --- | --- |
| Harada score | 0.591 | 0.533-0.648 | 83.5% | 34.6% | 30.5% | 85.9% |
| Kobayashi score | 0.592 | 0.529-0.656 | 30.4% | 88.1% | 46.7% | 78.7% |
| Egami score | 0.530 | 0.468-0.592 | 18.3% | 87.8% | 33.9% | 75.6% |
| Formosa score | 0.579 | 0.518-0.641 | 48.7% | 67.2% | 33.7% | 79.2% |
| Nomogram model | 0.790 | 0.742-0.837 | 79.1% | 68.4% | 46.2% | 90.5% |

AUC, area under the curve; CI, confidence interval; PPV positive predictive value, NPV negative predictive value.





Figure S1. Internal calibration curve of the nomogram for the prediction of the CAL.





Figure S2. Decision curve analysis of the nomogram
